# Smartphone applications for physical activity promotion from physical education

**DOI:** 10.1007/s10639-022-11108-2

**Published:** 2022-05-19

**Authors:** Francisco Javier Gil-Espinosa, Adriana Nielsen-Rodríguez, Ramón Romance, Rafael Burgueño

**Affiliations:** 1 Andalucía Tech, Faculty of Educational Sciences, IBIMA, Researching in Sport Sciences (RSS) Research Group, Universidad de Málaga, Campus de Teatinos s/n, 29010 Málaga, Spain; 2 Andalucía Tech, Faculty of Educational Sciences, Department of Didactics of Languages, Arts and Sports, Human Kinetics and Body Composition Laboratory, Universidad de Málaga, Campus de Teatinos s/n, 29010 Málaga, Spain; 3 Department of Education, University of Almeria, Almeria, Spain

**Keywords:** Mobile phone app, Educational technology, Digital literacy, Mobile device, Secondary school

## Abstract

Smartphone applications (apps) are thought to be an adequate instructional strategy not only to improve the quality of the teaching in physical education (PE), but also to effectively promote leisure-time physical activity (PA) of adolescent students in this context. Although the use of smartphone apps has been generalized in PE, little is known about the curricular approach of smartphone apps to be implemented by teacher to teach specific curricular contents in PE lessons. Therefore, the aim of this research was threefold: a) to conduct a systematic search for smartphone apps focused on PA and sport; b) to assess the features, content and quality of every included smartphone app; and c) to analyze the relationships between every selected app and the secondary PE curriculum. Systematic searches were completed on Google Play Store from January 2021 to March 2021. Apps were included when they met: main goal focused on PA and sport; permitted use by underage; they are free; user scores of at least 4. The app selection process was carried out by several reviewers and concordance measures were estimated. Additionally, an app quality assessment was independently conducted by three reviewers. A total of 18 apps focused on PA were included. Particularly, eight apps were suitable for fitness, health and quality of life curricular content; two for sports content; four for body expression content; and four apps for outdoor PA content. The mean quality score was 4.00. Apps could be helpful for teachers to implement the secondary PE curriculum and effectively promote PA among adolescent students.

## Introduction

The digital technology (e.g., digital blackboard, tablets, smartphone apps) has been incorporated as key elements into every sphere of our society such as health, work and education (Organisation for Economic Cooperation and Development, 2019). The use of digital technology to support the teaching and learning process has grown sharply in recent years (Sargent & Casey, 2020), and its implementation in education system has been accentuated by the COVID-19 pandemic. Indeed, Calderón-Garrido et al. (2022) review concluded that the scientific production seems to bet on the use of smartphones in class, as it is beneficial for educational objectives. In physical education (PE), while most research has focused on reporting usefulness and advantages derived from the use of digital technology in PE (Cushion and Townsend, 2019; Sargent & Casey, 2020), little attention has been paid to the question about whether there are smartphone apps that could be linked with PE curriculum to promote leisure-time physical activity (PA) in adolescents from the context of the secondary school PE. There is thus a need to help PE teachers take a curricular approach to the selection and implementation of smartphone applications (apps) that contribute to addressing the curriculum. This would allow us to recommend an improved use of smartphone apps for PE lessons, which would lead to improve the quality of the teaching and learning process with more meaningful learning experiences for students, and to accomplish one of the main educational goals, such as leisure-time PA promotion (SHAPE America– Society of Health and Physical Educators, 2014). This research sought to carry out a systematic search for smartphone apps focused on PA that are available in the Google Play. Next, the interplay between every included smartphone app and the secondary PE curriculum was further analysed.

### Secondary physical education curriculum

In common with another national PE curricula (e.g., SHAPE America– Society of Health and Physical Educators, 2014; Irish Proffessional Service for Teachers, 2022; British Council for the Curriculum, Examinations and Assessment, 2022), one the of the main curricular goals for PE, in Spain, is to develop students as physically literate people capable of applying the knowledge, skills and attitudes needed for lifelong health-related PA (Orden 15 of January, 2021; Royal Decree 1105/, 2014). In order to structure the knowledge, skills and attitudes to be learnt by students when completing secondary education, the content blocks of Spanish curriculum for secondary PE could be organized according four: a) fitness, health and life quality, which includes contents related to the development of basic physical capacities, the promotion of healthy lifestyles (e.g., regular PA, nutrition and adequate rest breaks) and avoidance of potential health-damaging behaviours (e.g., sedentarism, tobacco, alcohol and drugs), as well as the identification of health benefits derived from a good level of fitness and regular leisure-time PA; b) games and sports, which comprises contents referring to the understanding of games and sports as sociocultural phenomena, the knowledge of Spanish traditional games, as well as the development of technical and tactical skills, the knowledge and respect for the rules of individual and team sports; c) body expression, which brings together contents concerning creative and artistic communication and emotional regulation using body (e.g., dance, choreography, and dramatization) d) outdoor PA, which tackles contents regarding the interaction between students and nature through PA (e.g., hiking, orientating, and outdoor challenges), and the understanding and appreciation of the natural environment.

The Spanish curriculum for secondary PE states two 60-minute lessons per week, despite the substantial body of evidence indicating PE helped students meet daily PA recommendations (Kerr et al., 2018; Kwon et al., 2020; Lee & Gao, 2020). Thus, there is a need for teachers to develop and implement instructional strategies aiming to promote students’ levels of leisure-time PA from PE lessons. For this goal, the use of digital technology, including smartphone apps, is recommended not only to develop key digital competence, but also to improve the comprehensive education of students in general, and promote leisure-time PA from the context of the secondary school PE in particular (Orden 15 of January, 2021; Royal Decree 1105/, 2014).

### Smartphone apps in physical education

The use of digital technology in PE is considered as a relevant instructional recourse given its potential in supporting the teaching and learning process in a manner that fits the nature of PE (Casey et al., 2017). For instance, digital technology such as digital blackboard, tablets, video cameras, electronic devices, and smartphone apps provide students with opportunities to reinforce their learning, and to foster their autonomy (Hyeonho & Taemin, 2021). Recently, inquiring app-integrated PE in which several apps are utilized on the smartphone in PE has been on the rise to ease the teaching learning process (Krause & Sanchez, 2014; Zhu & Dragon, 2016). PE experts have suggested smartphone apps perform different roles in improving the quality of PE: a) they can serve as communicational tools such as scoreboard, whiteboard or display platforms; b) they can serve as classroom management tools by being useful for timers, music displayers, and microphones; c) they can be used as tools for information delivery, feedback, lesson plans, assessment; and d) they should be personalised based on every student’s need and skills (Goodyear et al., 2019; Penney et al., 2012; Pyle & Esslinger, 2014; Sinelnikov, 2012).

It is, thus, thought to develop students as physical literate people via smartphone apps may represent an effective and innovative strategy to promote leisure-time PA from the context of the secondary school PE. This idea relied on the fact that most secondary students have a generalised use of digital technology in their everyday life through the utilisation of social networks, websites, blogs, and smartphones, and user accounts for several apps (Böhm et al., 2019; Direito et al., 2015; Goodyear & Armour, 2021; Vega-Ramírez et al., 2020).

### Literature review on apps in physical education

The digital technology could allow a greater variety of instructional strategies in PE while helping to develop in students attitudes, knowledge and behaviours for a more physically active life. Therefore, teachers need to experiment with apps related to the PE teaching-learning process (Yu et al., 2018). Accordingly, previous research about the use of apps, conducted in the school context of PE, has evidenced advantages in motivation, knowledge of results, assessment, and the improvement of student autonomy, among others (Kerner & Goodyear, 2017; Klenk et al., 2017; Phillips et al., 2014; Vega-Ramírez et al., 2020). Schwartz and Baca (2016) point out that many PA apps are based on behavioural theory and use elements of gamification for success, with personal goals and specific feedback. Lee (2018) recommends using apps to facilitate students’ group activities, as well as the knowledge of results. Similarly, a growing body of research has pointed out that the implementation of smartphone apps was an efficient instructional strategy to increase in-classroom PA, as well as to promote leisure-time PA among adolescent students in PE (Böhm et al., 2019; Brickwood et al., 2019; Gil-Espinosa et al., 2020; Lau et al., 2011).

Likewise, Mokmin and Jamiat (2021) designed a mobile application taking into consideration the motor learning theory (Muratori et al., 2014) and Mayer’s cognitive theory of multimedia learning (Mayer, 2011), obtaining satisfactory results in motivation and PA performance after its implementation with students. In turn, the results of Papastergiou et al. (2021) research with primary school students conclude that the use of apps improved their interest, enjoyment, and motivation, while teachers had more time to provide individualised feedback. Similarly, research carried out by Yu (2020) in secondary education found that, through the use of mobile applications, students improved their tactics and performance of badminton skills while allowing for more active learning and improved knowledge of the results, concluding that PE teachers can integrate the use of mobile applications in their teaching work.

In any case, the educational benefits of using mobile applications in PE will be determinate by the design of the learning activities (Greve et al., 2022), which is why their link with the curriculum is crucial. PE teachers are recommended to align the selection of apps with the PE learning goals (Lee & Gao, 2020). Krause et al. (2020) recommend increasing research on the use of technology in PE, as well as improving the training of PE teachers in the integrated use of technology in the subject.

### The current research and research question

Smartphone apps represent a potential means both to develop students as physical literate, and to promote leisure-time PA from the context of the secondary school PE. In accordance with previous research, the number of smartphone apps, particularly those related to PA and sports, has been on the Google Play (Arigo et al., 2020). This great quantity of smartphone apps represents a good opportunity to provide students with improved meaningful learning experiences, yet it also makes it quite difficult for teachers to select and implement the most suitable app in accordance with curricular content to be taught in lessons. Although previous research has well documented the benefits from the use of smartphone apps on learning-related outcomes in students such as increased levels of in-classroom and leisure-time PA, the question about the curricular approach to smartphone apps to be used by teachers to teach specific curricular contents in the classroom remains still to be examined. To the best of our knowledge, no studies were found to analyse smartphone apps to promote PA in the school context and its potential relationships to the curriculum and possible use by PE teachers. At the same time, Koekoek and van Hilvoorde (2018) thought over the need to understand the way teachers select digital technology, without losing educational goals. Further, this question becomes even more important due to the great use of digital technology in general, and particularly smartphone apps in PE to adapt the implementation of curriculum to the new instructional models derived from the COVID-19 pandemic (López-Fernández et al., 2021).

This research attempts to provide a response to the questions about which smartphone apps on PA and sports are linked directly to secondary curriculum for PE, and how PE teachers might implement them to teach curricular content with the purpose of promoting leisure-time PA from the school context of PE. Therefore, the aim of this research was threefold. The first objective was to carry out a systematic search for smartphone apps focused on PA and sport that are available in the Google Play. The second objective included a quality assessment for every smartphone app included in this study. The third objective consisted of the analysis of the relationship between every smartphone app and the PE curriculum, as well as the provision of possible strategies for the implementation of each app.

## Materials and methods

### Search strategy

Systematic searches were individually completed by two researchers (R1 and R2) in Google Play Store between 12th January 2021 and 31st March 2021. The identification of apps was performed using the following search terms: “physical education”, “physical activity”, “health”, “fitness”, “sport”, “body expression”, “corporal language”, “dance”, “outdoor physical activity”. Search terms were entered into the Google Play Store in isolation or in different combinations based on Boolean logic (i.e., AND, NOT, OR).

### Inclusion criteria and selection process

The apps selection process was developed in two phases. In the first phase and after removing duplicates, apps were retrained and registered in a database when they met the following criteria: a) they were written in Spanish or English (description and application), and b) they had the main goal focused on PA promotion. Apps could be used in isolation or in combination with an external device (e.g., PA tracker) or a back-office system, for instance, to communicate with a PE or PA professional. This first phase was carried out by two reviewers (R3 and R4) who individually evaluated the names and descriptions of the apps against these two criteria using a checklist (response: yes, or not). To solve disagreements, a consensus meeting was held between both reviewers and the main author of this research. The apps identified in this first phase were, then, included in the second selection phase. In this phase, a second set of inclusion criteria was established to identify the apps that would be included in this study. They would be included when: a) their use is allowed to underage; b) they are free (Bearne et al., 2020; Simões et al., 2018); c) user score of at least 4 (scale range 1 to 5) following previous app reviews (Bearne et al., 2020; Simões et al., 2018); d) a number of user scores equal to 100 or higher (Bearne et al., 2020; Simões et al., 2018). Two reviewers (R5 and R6) individually evaluated the names and descriptions of the apps against the established inclusion criteria using a checklist (answer: yes, or not). A consensus meeting was held to resolve disagreements between both reviewers with the help of the main author of this research.

### Quality assessment

In line with previous app reviews, an app quality assessment was completed through the Mobile App Quality Ratings (MARS) (Stoyanov et al., 2015). It includes 23 items divided into 5 dimensions: engagement (5 items, e.g., “interest”), functionality (4 items, e.g. “ease of use”), aesthetics (3 items, e.g. “Visual appeal: How well does the app look?”), information quality (7 items, e.g. “accuracy of app description”), and subjective quality (4 items, e.g. “Would you recommend this app?”) (Stoyanov et al., 2015). Every item is answered on a 5-point Likert scale ranging from 1 (inadequate) to 5 (excellent). To gather quality evidence for each app, a global average score from the mean of each MARS dimension was estimated (Stoyanov et al., 2015). This assessment was independently carried out by three reviewers (R1, R3 and R6). Likewise, disagreements were solved by a consensus meeting between the reviewers and the main authors of this study.

### Relationships between apps included and PE curriculum

Once apps were included in this research, the authors individually downloaded the different apps and proceeded to individually analyse them by taking into consideration the four content blocks (i.e., fitness, health and life quality; games and sports; body expression; and outdoor physical activities), established after the analysis of the Spanish secondary PE curriculum (Orden 15 of January, 2021; Royal Decree 1105/, 2014), and its respective implementation in the classroom. Subsequently, each author proposed a series of possible practical applications for every app. Lastly, a meeting was held to agree both points and to resolve disagreements among the authors.

### Data analysis

For categorical data from a checklist, the degree of agreement among reviewers was computed using the kappa (κ) index. Consistent with Landis and Koch (1977), there is an insignificant agreement with values below 0.20, small with values between 0.21 and 0.40, moderate with values between 0.41 and 0.60, acceptable with values between 0.61 and 0.80, and excellent with values between 0.81 and 1.00. For continuous data from MARS scores, the degree of agreement between reviewers was estimated by the intraclass correlation coefficient (ICC, model: bidirectional mixed effects, absolute agreement). According to Koo and Li (2016), there is a poor agreement when values are less than 0.50, moderate when values are between 0.50 and 0.75, good when values are between 0.75 and 0.90, and excellent when values are greater than 0.90.

## Results

### App selection process

**Fig. 1:**
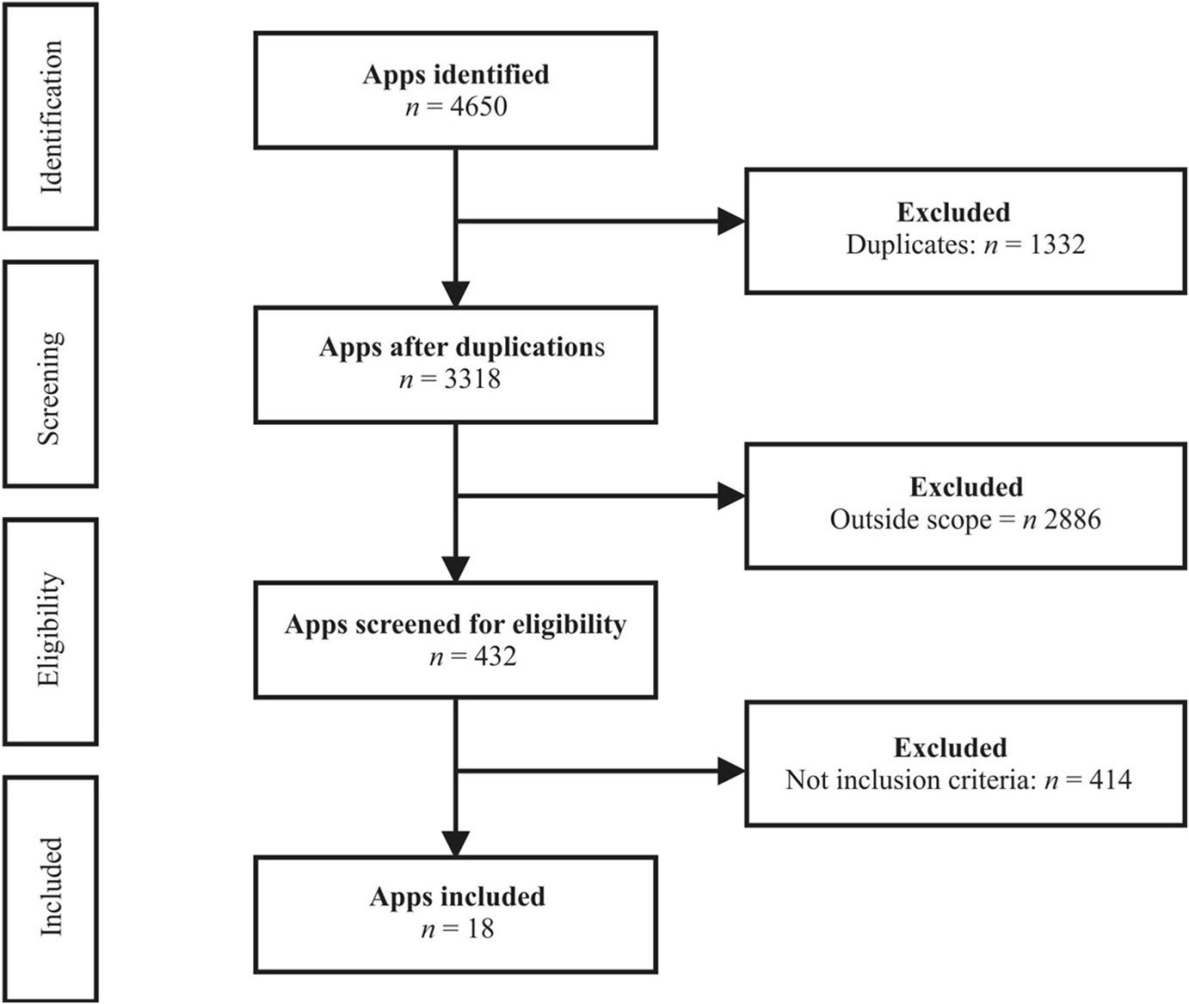
Flow diagram displaying the app selection process

Figure 1 shows a flow diagram for the app selection process. A total of 4650 apps in Google Play Store were initially identified. After removing duplicates (κ = 1.00) and out-of-scope apps (κ = 0.95), 432 app titles and descriptions were screened for eligibility based on a set of inclusion criteria (κ ranging from 0.85 to 1.00). Once this app selection process was completed, 18 apps were definitively included in this study.

### App quality

The total MARS mean score was 4.00 (SD = 0.50) out of 5 (ICC = 0.90, 95%CI = 0.80–1.00) for the totally of apps included in this research. The dimension with the highest score was subjective quality (M = 4.30, SD = 0.50; ICC = 0.89, 95%CI = 0.80–0.97), followed by functionally (M = 4.25, SD = 0.25; ICC = 0.85 95%CI = 0.76–0.94), aesthetics (M = 4.20, SD = 0.20; ICC = 0.90, 95%CI = 0.79–0.95), information quality (M = 3.75, SD = 0.70; ICC = 0.89, 95%CI = 0.80–0.97), and engagement (M = 3.70, SD = 0.60; ICC = 0.81, 95%CI = 0.76–0.88).

### Links with the secondary PE curriculum

The 18 apps that met the inclusion criteria were organized according to the four content blocks present in the Spanish secondary PE curriculum: 1) fitness, health and life quality, 2) games and sports, 3) body expression, and 4) outdoor PA (Orden 15 of January, 2021; Royal Decree 1105/, 2014). A description of each of them is shown, as well as proposals for possible applications to PE, which should be understood as ideas that teachers must adapt to variables such as the characteristics of their students and schools, among others.

#### Apps for fitness, health and life quality

Table 1 yields eight apps to be used in PE for the curricular contents related to fitness, health and life quality. These apps would allow students and/or teachers to create individual and collective physical challenges, to encourage them to prepare exercise for the group-class or to work with other subjects in an interdisciplinarity manner.

**Table 1:** Apps linked to the fitness, health and life quality curriculum content block

Fitness, health and life quality		
App	Description	Possible practical applications in PE^(*)^
Adidas Runtastic	Develops activity tracking apps and services such as training logs, data analysis, comparisons to other users, and other functions to help users improve their overall fitness	Creation of individual or collective challenges. Interdisciplinarity with subjects such as Geography and history and biology, among others. Encourage the autonomy of the students by facilitating them to prepare the exercises and physical activity and then share it with the rest of the group-class.
Gowod	Allows to test and improve the mobility and flexibility	
Mapmyrun	Know your distance, pace, calorie burn, elevation, and more	
Realfooding	Food database, food scan, food log, user community	
Smartwod generator	Workouts to choose based on the equipment available	
Strava	Internet service for tracking human exercise which incorporates social network features. It is mostly used for cycling and running using GPS data	
Stretching & Flexibility at home	Allows you to know popular stretching exercises	
Sworkit	Allows to customize and play personalized video workouts	

^(*)^Based on (Cabrera Ramos et al., 2019; Cummiskey, 2011; Gil-Espinosa et al., 2020; Mokmin & Jamiat, 2021; Papastergiou et al., 2021; Vega-Ramírez et al., 2020)

#### Apps for games and sport

Table 2 shows the two apps selected for games and sports contents in PE. Both apps would enable students to organise competitions and to create teams for sports activities. In addition, the two apps would facilitate interdisciplinary work with other subjects.

**Table 2:** Apps linked to the games and sports curriculum content block

Games and sports		
App	Description	Possible practical applications in PE^(*)^
Leverade real play	Organisation of team sports activities	Allows students to be empowered by organizing competitions. Interdisciplinarity with subjects such as mathematics.
Team maker	Creation of groups and teams	

^(*)^Based on (Andre & Hastie, 2018; Cecchini and Carriedo, 2020; Díaz, 2020; Vega-Ramírez et al., 2020)

#### Apps for body expression

Table 3 displays four apps to address the curricular contents of body-expression in PE. These apps emphasise the possibility of creating choreographies, rhythms and characterisations autonomously. Furthermore, they would allow students to work on creative projects.

**Table 3:** Apps linked to the body expression curriculum content block

Body expression		
App	Description	Possible practical applications in PE^(*)^
Acrosport eps	Pre-made body figures and involve students in a creation process	Learning application based on creative projects, choreographic compositions, rhythm, characterizations, among others.
Just dance	Songs and choreographies without the need for a game console	
TikTok	Is a video-sharing social networking service	
Youcam makeup	Try makeup trends with live camera. Edit selfies with retouching	

^(*)^Based on (Bodsworth & Goodyear, 2017; Gorman, et al. 2019; Martínez, 2019; Phelps et al., 2021)

#### Apps for outdoor physical activities

Table 4 shows the four apps that can be used in order to address outdoor physical activities from a curricular perspective in PE. They would allow students to organise and conduct sports events in natural and/or urban environments, in addition to facilitating work with other subjects in an interdisciplinary way.

**Table 4:** Apps linked to the outdoor PA curriculum content block

Outdoor physical activity		
App	Description	Possible practical applications in PE^(*)^
Geocaching	It is based on hiding and finding “treasures” (objects left by users) with the help of GPS	Carrying out tours on the natural or urban environment. Interdisciplinarity with other subjects such as geography and history, biology, mathematics, physics and chemistry. Calculation of scales and mathematics.
QR scanner	Decode QR codes directly	
Maps	Navigate with GPS in real time	
Munzee	Game based on geolocation, that is, through locations via GPS	

^(*)^Based on (Gallego-Lema et al., 2017; Jansson et al., 2019; Michalakis et al., 2020; Ruiz et al. 2020)

## Discussion

The objective of this research was threefold. The first of them was to carry out a systematic search for smartphone apps focused on PA and sport that are available in the Google Play. The second objective included a quality assessment for every smartphone app included in this study. The third objective consisted of the analysis of the relationship between every smartphone app and the PE curriculum, as well as the provision of possible strategies for the implementation of each app.

The main results revealed a total of 18 smartphone apps included: eight apps were suitable for fitness, health and quality of life curricular content; two for games and sports content; four for body expression content; and four apps for outdoor PA content. The mean quality score was 4.00. This research contributes to the scientific literature a list of apps that, linked to curriculum blocks of PE content, could help to improve their educational use by PE teachers, consistent with Almusawi et al. (2021), who concluded that PE teachers need technology to save class time, making it more convenient for PE. A second contribution of the research is the design of possible practical applications in PE of the apps, linked to each of the curricular contents, based on the opinion of PE teachers and the review of the scientific literature. This study expands on literature by shedding the need to link the use of apps to the school curriculum and its contents.

When we refer to the educational context, teachers must be very demanding with the criteria for selecting the apps to be used. Of the 4650 apps initially identified, only 18 were selected for possible application in the educational context. This suggests a proliferation and offer of apps that, although they might seem useful in the educational process, for various reasons are not recommended. Of the 18 selected apps, eight have been linked to the “fitness, health and life quality” content block, two to “games and sport”, four to “body expression” and four to “outdoor physical activities”. This could be motivated by the creation of these apps for society in general and not specifically for the educational context and, therefore, the most of them are related to physical condition, health and quality of life.

Therefore, it would be advisable to involve and coordinate educational administrations in order to design apps for educational purposes, both in collaboration with teachers and with companies specializing in new technologies. In this way, compliance with the inclusion criteria that are determined adequate would be guaranteed and the work of the teaching staff would be facilitated. In this vein, Almusawi et al. (2021) suggests a focus on readiness encourages collaboration between sectors and industries to bring out the best innovative solutions for PE, as well as the collaborations of different Ministries in the app design. Just as the administration, for example, coordinates, controls and finances the textbooks to be used, one should start thinking about a similar implication with new technologies. The quality of apps designed for children and adolescents (evaluation of commitment and quality of information) correlates with the number of techniques identified to change health behaviors (Ng et al., 2019). Another interesting variable to be considered in the design of the apps is that the interest in the use of mobile devices can have a novel effect that later disappears (Ridgers et al., 2018). However, initial motivation could serve to establish individual awareness of PA levels. PE teachers must assess the apps, prior to use with students, so that they comply with the provisions of Organic Law 3/, 2018, on the Protection of Personal Data and guarantee of digital rights, while also adjusting to the proposed objectives and the students involved. In minors, request authorization from legal guardians for use by students.

Teachers are ultimately responsible for making the decision regarding the selection and use of the apps, recommending a group or collective involvement of the students, whenever possible, in order to avoid isolation. A possible strategy could be to provide challenging activities such as defiant, seeking self-efficacy, motivation and that stimulate social support (Martins et al., 2016), paying special attention to promoting the adolescent’s perception of competence and positive experiences (Martins et al., 2018). In fact, presenting collective challenges has proven to be an effective methodological strategy (Gil-Espinosa et al., 2020). Thus, physical activities that promote social relationships or include team activities are more likely to be successful (Johansson & Ruud, 2016). Furthermore, combining school interventions with family or community participation appears to be an effective strategy (Parody et al., 2019). It is important to consider variables such as whether or not they are free, the minimum age for registration and use, the need for authorization from legal guardians, the authorization of the educational center for the use of personal mobile devices or the existence of advertising in the apps, among others.

In any case, it is essential that teachers frame the use of apps for educational purposes such as increasing the students’ motivation towards PA, the implementation of more students-centered models or the work of digital competence, as some investigations have already concluded (Gil-Espinosa et al., 2020; Zhao et al., 2016). In this vein, Lau et al. (2011), in their review, provide evidence supporting the positive effects of the use of digital technology in PA interventions for children and adolescents.

Undoubtedly, the integration of the use of new technologies by students should guide us to work towards a responsible, formative, safe and educational use of them. In the case of PE, it is essential to guide students towards apps based on adequate scientific and technical criteria. Therefore, teachers must seek to empower students in the digital information age and, for this, they must have sufficient training to integrate the use of digital technology in learning (Goodyear & Armour, 2018; Pereira et al., 2019). This is how the Spanish curriculum establishes it in secondary education (Royal Decree 1105/, 2014).

Therefore, taking into consideration that PE time during school hours is insufficient, the increase of PA levels of students during non-school hours should be promoted (Gil-Espinosa et al., 2020). In this context, the use of apps is presented as a resource to promote strategies that increase adolescents’ PA and health (Böhm et al., 2019; Cummiskey, 2011; Dute et al., 2016; Simões et al., 2018). Likewise, Lee and Gao (2020) concluded that teachers are recommended to align the use of apps with the PE learning goals. Indeed, Cheng and Chen (2018) argued that the combination of traditional and a mobile APP support learning system is an effective approach that would help students to improve their health-related fitness achievements.

The strengths of this study include the systematic search for apps from Google Play, the use of the MARS instrument to assess quality and its relationships with the PE curriculum in secondary education. Another strength is the participation of PE teachers of Secondary Education and University. Further, app ratings were performed by reviewers and other ones participated in different phases. One highlight of this research is that digital technology can be integrated with the PE curriculum to improve the teaching-learning process and promote PA among students. Future research should test the overall effectiveness of apps designed to promote PA among adolescents considering its link with the official curriculum. Finally, research examining the accuracy of app content and developer expertise should also be of high priority.

The limitations of this study include the exclusion of apps with low interrater reliability for the scoring of app quality through the MARS scale. This may be due to the subjective nature of some sections of the scale. The temporal relevancy of the results from this study may also be considered a limitation. Another limitation was the short period used for the individual assessment of each app. Likewise, it would be convenient to carry out similar research in the App store.

## Conclusions

The results suggest that popular apps for measuring and, potentially, promoting PA are of moderate quality if we look at its use in the school context. The app content quality, particularly the use of international guidelines on linking PA to the official curriculum could be improved. Furthermore, based on the findings from this assessment, we suggest that education administrators and teachers should be involved in the development of apps targeting student’s PA behaviors; that apps should be developed considering the target group (aged, motivation…) and the respective PA recommendations established by the WHO (2020); and consider the official curriculum.

More effort is needed to incorporate components that are more likely to influence PA behavior change, which is ultimately what they were developed for, while maintaining good functionality. Educational administration and developers should collaborate to provide self-monitoring and social comparison in students to enhance a comprehensive, competency-based education. In summary, this study seeks to advance research on the use of apps in PE, focusing on its link with the curriculum contents.

## Data Availability

The dataset generated for this study is available on request to the corresponding author.
